# Neutrophil glucose flux as a therapeutic target in antiphospholipid syndrome

**DOI:** 10.1172/JCI169893

**Published:** 2024-06-13

**Authors:** Ajay Tambralli, Alyssa Harbaugh, Somanathapura K. NaveenKumar, Megan D. Radyk, Christine E. Rysenga, Kaitlyn Sabb, Julia M. Hurley, Gautam J. Sule, Srilakshmi Yalavarthi, Shanea K. Estes, Claire K. Hoy, Tristin Smith, Cyrus Sarosh, Jacqueline A. Madison, Jordan K. Schaefer, Suman L. Sood, Yu Zuo, Amr H. Sawalha, Costas A. Lyssiotis, Jason S. Knight

**Affiliations:** 1 Division of Rheumatology, Department of Internal Medicine,; 2 Division of Pediatric Rheumatology, Department of Pediatrics,; 3 Department of Molecular and Integrative Physiology, and; 4 Division of Hematology and Oncology, Department of Internal Medicine, University of Michigan, Ann Arbor, Michigan, USA.; 5 Departments of Pediatrics, Medicine, and Immunology, and Lupus Center of Excellence, University of Pittsburgh School of Medicine, Pittsburgh, Pennsylvania, USA.

**Keywords:** Autoimmunity, Autoimmune diseases, Glucose metabolism, Neutrophils

## Abstract

Neutrophil hyperactivity and neutrophil extracellular trap release (NETosis) appear to play important roles in the pathogenesis of the thromboinflammatory autoimmune disease known as antiphospholipid syndrome (APS). The understanding of neutrophil metabolism has advanced tremendously in the past decade, and accumulating evidence suggests that a variety of metabolic pathways guide neutrophil activities in health and disease. Our previous work characterizing the transcriptome of APS neutrophils revealed that genes related to glycolysis, glycogenolysis, and the pentose phosphate pathway (PPP) were significantly upregulated. Here, we found that neutrophils from patients with APS used glycolysis more avidly than neutrophils from people in the healthy control group, especially when the neutrophils were from patients with APS with a history of microvascular disease. In vitro, inhibiting either glycolysis or the PPP tempered phorbol myristate acetate– and APS IgG–induced NETosis, but not NETosis triggered by a calcium ionophore. In mice, inhibiting either glycolysis or the PPP reduced neutrophil reactive oxygen species production and suppressed APS IgG–induced NETosis ex vivo. When APS-associated thrombosis was evaluated in mice, inhibiting either glycolysis or the PPP markedly suppressed thrombosis and circulating NET remnants. In summary, these data identify a potential role for restraining neutrophil glucose flux in the treatment of APS.

## Abstract 2

Inhibition of glycolysis or the pentose phosphate pathway to restrain glucose flux, which is increased in patients with antiphospholipid syndrome, reduces thrombosis in mice.

## Introduction

Antiphospholipid syndrome (APS) is a thromboinflammatory autoimmune disease that is characterized by the presence of persistently positive antiphospholipid (aPL) antibodies measured by immunoassays for anticardiolipin and anti-β-2-glycoprotein I (anti-β_2_GPI) antibodies or by the lupus anticoagulant functional screen, which detects a variety of aPL antibody species including anti-phosphatidylserine/prothrombin (anti-PS/PT) antibodies (1, 2). APS is best known for increasing the risk of thrombotic events and late-term pregnancy morbidity and is usually treated with lifelong anticoagulation (1, 3). However, anticoagulation is incompletely effective for many patients with these manifestations (4). Further, 20% of patients with APS will develop microvascular complications, such as diffuse alveolar hemorrhage (DAH), renal thrombotic microangiopathy (TMA), and catastrophic APS (CAPS), which are not adequately treated with anticoagulation (5–7).

Neutrophil extracellular traps (NETs) are web-like tangles of decondensed chromatin complexed with granule-derived proteins (8) that are released in response to infectious organisms and sterile mediators (9). NETs have been shown to influence many autoimmune and thromboinflammatory diseases, including systemic lupus erythematosus (SLE), rheumatoid arthritis, APS, and COVID-19 (10–14). NETs contribute to endothelial damage (15), autoantibody formation (16, 17), and thrombosis (18, 19). Neutrophils from patients with APS have a lower threshold for spontaneous neutrophil extracellular trap release (NETosis) (18). In APS mouse models, impairing NETosis by depleting neutrophils (19), administering intravenous deoxyribonuclease (19), potentiating adenosine A_2A_ receptor signaling (20), or boosting cyclic AMP levels (21) is protective against aPL antibody–mediated pathology. Further, total IgG fractions isolated from patients with APS trigger Fc receptor–independent NETosis from healthy neutrophils by engaging neutrophil Toll-like-receptor and β-integrin signaling (18, 22, 23).

As the first immune cells at sites of inflammation, neutrophils have significant energy requirements and must rapidly access adenosine triphosphate (ATP) to enable their functions. As such, the historical focus was especially on their use of glycolysis, which provides faster access to energy than other metabolic processes (24–27). However, accumulating evidence suggests that neutrophils can also leverage other pathways, including the pentose phosphate pathway (PPP), fatty acid oxidation (FAO), the tricarboxylic acid (TCA) cycle, and oxidative phosphorylation (OXPHOS) for their various activities, such as differentiation, chemotaxis, and degranulation (28). To generate microbe-neutralizing reactive oxygen species (ROS) in the form of an “oxidative burst,” neutrophils assemble the NADPH oxidase complex (NOX) at plasma and phagosome membranes. As its name suggests, NOX is dependent on NADPH generation, which, in neutrophils, is primarily mediated by the PPP (29–33). Whereas NETosis seems to require ROS in all contexts, various triggers of NETosis differ in their relative utilization of NOX-mediated versus mitochondrial ROS (34, 35). Moreover, only a few studies have analyzed the metabolic requirements for NETosis, mainly in the setting of stimulation with the potent NOX activator, phorbol myristate acetate (PMA), which has been found to require glycolysis and the PPP (36–39). The importance of the PPP has also been noted in the clinic, where patients with severe glucose-6-phosphate dehydrogenase (G6PD) deficiency have reduced NETosis (40–42).

In this study, we sought to identify the metabolic determinants driving the hyperactivity of APS neutrophils. We identified that neutrophils from patients with APS, especially those with a history of microvascular complications, use glycolysis more avidly. We also found that glycolysis and the PPP are key metabolic pathways required for the execution of APS-mediated NETosis. Further, pharmacologic inhibition of either glycolysis or the PPP reduced NETosis and APS-associated thrombosis in mice. Collectively, our data suggest that reprogramming glucose metabolism could be an effective strategy to restrain neutrophil hyperactivity and reduce the risk of thrombosis in APS.

## Results

### Metabolic pathways are altered in APS neutrophils.

Via RNA-Seq, we recently compared the transcriptome of primary APS neutrophils to those of age- and sex-matched controls and found a prominent metagroup for metabolic processes (43). Further gene ontology analysis derived from the R package TopGo, which was performed through the R/Bioconductor package FGNet (44), identified that these genes were especially involved in glucose oxidation, glycogenolysis, and the PPP (Table 1). Individual genes increased in these pathways include *CALM2* (2.10-fold, *P* = 2.11×10^–5^), *GYG1* (2.01-fold, *P* = 2.8×10^–6^), *MGAM* (2.37-fold, *P* = 6.63×10^–6^), *NUDT5* (2.26-fold, *P* = 1.82×10^–4^), *PGM1* (2.00-fold, *P* = 4.99×10^–6^), *PPP1R3D* (2.05-fold, *P* = 3.87×10^–4^), *PYGL* (2.08-fold, *P* = 5.91×10^–6^), *TALDO1* (2.68-fold, *P* = 2.31×10^–7^), and *TKT* (2.34-fold, *P* = 1.54×10^–7^) (43). Other relevant metabolic genes that tended to be increased include *HK1* (1.29-fold, *P* = 3.30×10^–2^) and *G6PD* (1.56-fold, *P* = 5.62×10^–4^).

### APS neutrophils have increased glycolysis usage.

To better understand the bioenergetics of APS neutrophils, we used metabolic flux analysis to profile neutrophils from (a) healthy controls, (b) patients with APS, (c) patients with circulating aPL antibodies without criteria-defining APS (i.e., “aPL-only” because they have no history of thrombosis or pregnancy morbidity), or (d) patients with thrombosis for reasons other than APS (i.e., “thromb (aPL–)”). The demographic and clinical information for all participants are listed in Supplemental Table 1; supplemental material available online with this article; https://doi.org/10.1172/JCI169893DS1

**Figure 1:**
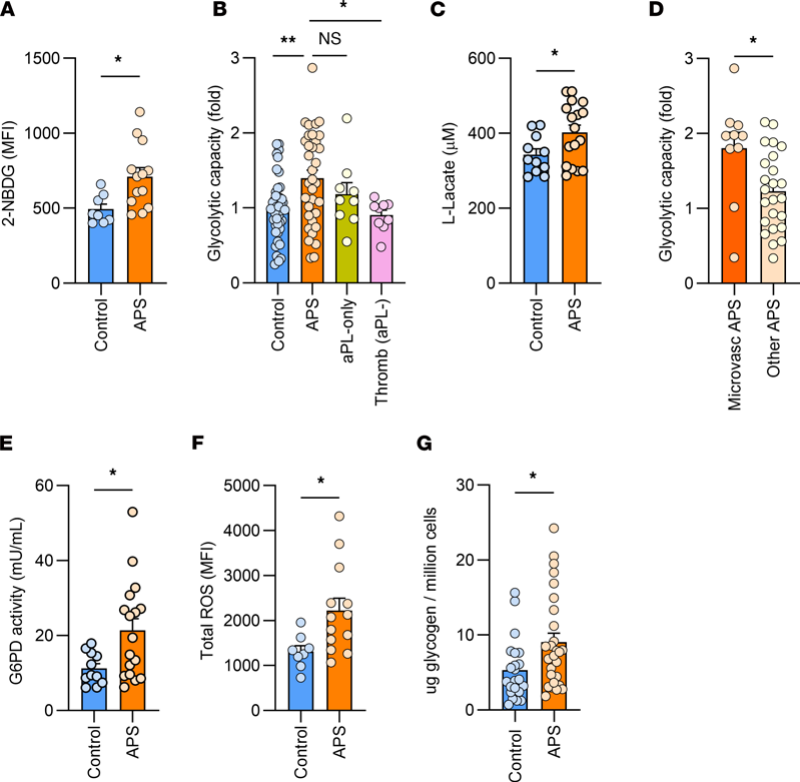
Metabolic parameters in neutrophils from people in the control group, patients with APS, patients with aPL-only, and patients with Thromb (aPL–). (**A**) 2-NBDG fluorescence in people in the control group (*n* = 8) and patients with APS (*n* = 13) by flow cytometry; **P < 0.01 using *t* test. (**B**) Extracellular flux analysis of neutrophils using the glycolysis stress test. Glycolytic capacity was defined as the extracellular acidification rate measuring the maximal cellular utilization of glycolysis. Presented as fold change compared with controls; *P < 0.05, **P < 0.01 using 1-way ANOVA with Holm-Šidák’s multiple comparison test. *n* = 42 controls, 34 APS, 9 aPL-only, and 9 Thromb (aPL–). (**C**) L-Lactate in cell culture supernatant from controls (*n* = 11) and patients with APS (*n* = 17); *P < 0.05 using *t* test. (**D**) Glycolytic capacity of patients with APS with a history of microvascular disease (defined as having a history of diffuse alveolar hemorrhage, thrombotic microangiopathy, or catastrophic APS, *n* = 10) as compared with patients with APS without these features (*n* = 24); *P < 0.05 using *t* test. (**E**) Intracellular G6PD enzyme activity from controls (*n* = 11) and patients with APS (*n* = 17); *P < 0.05 using *t* test. (**F**) Total cellular ROS production in people in the control group (*n* = 8) and patients with APS (*n* = 13) as measured with DCFDA fluorescence by flow cytometry; **P < 0.01 using *t* test. (**G**) Glycogen stores in controls (*n* = 22) and patients with APS (*n* = 27); *P < 0.05 using *t* test.

Using flow cytometry, we observed that 2-NBDG fluorescence was increased in patients with APS compared with controls (Figure 1A). However, we did not observe a difference in surface GLUT-1 expression between the 2 groups (data not shown). Using the Agilent Seahorse extracellular flux analyzer’s glycolysis stress test, we found that APS neutrophils had increased glycolytic capacity (the maximal cellular utilization of glycolysis) compared with people in the control group and patients with thromb (aPL–) (Figure 1B). There was also a numerical but not statistically significant increase in the glycolytic capacity in patients with APS compared with patients with aPL-only (Figure 1B). The patients with aPL-only were also not statistically different from controls, suggestive of an intermediate phenotype between patients with APS and the control group. This relationship between patients with APS and the other groups was maintained for basal glycolysis measurements as well (data not shown). As another readout of glycolytic flux, we found that APS neutrophils released more lactate than controls (Figure 1C).

We next sought to identify any relationships between neutrophil metabolic parameters and routine clinical markers of inflammation. Using the Pearson correlation coefficient, the glycolytic capacity of APS and aPL-only neutrophils had a positive association with the absolute neutrophil count (*r* = 0.4, *P* = 0.018). There was also a positive, but not statistically significant, association with C-reactive protein (*r* = 0.24, *P* = 0.18) and erythrocyte sedimentation rate (*r* = 0.34, *P* = 0.06). In summary, these data reveal that APS neutrophils have an increased ability to engage glycolysis, which associates with increased circulating neutrophil counts.

### Microvascular APS neutrophils have the highest glycolysis usage.

We were interested in whether metabolic parameters might help differentiate patient subsets. To assess this, we separated patients with APS based on whether they had ever had venous thrombosis, arterial thrombosis, an obstetric event, or a microvascular event (defined as having had at least 1 of DAH, TMA, or CAPS). The glycolytic capacities were similar in neutrophils from patients with APS with venous, arterial, or obstetric APS compared with patients without those features (data not shown). However, patients with APS with a history of microvascular events had an increased glycolytic capacity compared with those who did not have this history (Figure 1D). Of note, 5 of the 10 patients included in the microvascular group also had a history of macrovascular thrombosis. Statistically significant differences were not observed when we separated patients based on the usage of hydroxychloroquine or immunosuppressive agents (data not shown). In summary, when patients with APS were differentiated based on their clinical manifestations or medication usage, the subset of patients with APS with microvascular complications appeared potentially distinct from other patients with APS based on neutrophil glycolytic capacity.

### APS neutrophils have increased G6PD activity and total ROS production.

We measured the G6PD enzyme activity in neutrophil lysates and observed that lysates from patients with APS had approximately double the enzymatic activity of control lysates (Figure 1E). Enhanced PPP flux was also supported by increased total ROS production in APS neutrophils compared with controls (Figure 1F).

### APS neutrophils have increased glycogen content.

We found that APS neutrophils had increased glycogen compared with controls (Figure 1G). We also found a positive association between neutrophil glycolytic capacity and their glycogen content (*r* = 0.37, *P* = 0.0084).

### APS IgG-induced human NETosis relies on glycolysis and the PPP.

**Figure 2:**
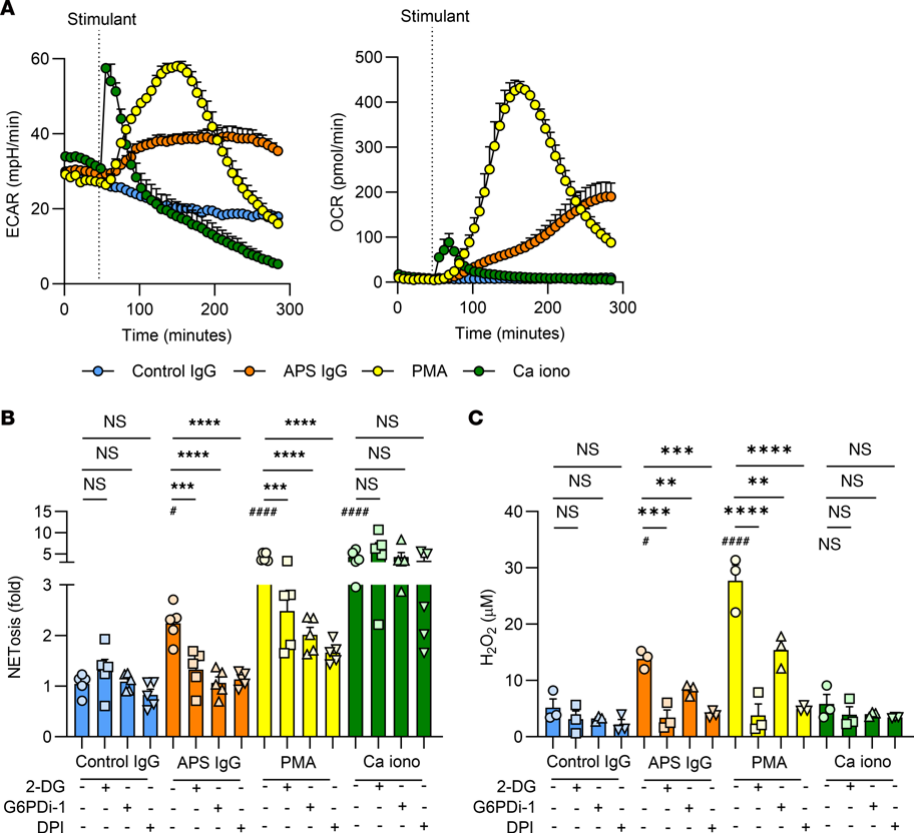
Glycolysis and the PPP are required for APS IgG–induced human neutrophil NETosis and ROS production. (**A**) Using a metabolic flux analyzer, neutrophils from controls were treated with the indicated stimuli, and ECAR (left) and OCR (right) trends were measured over 4 hours. These data are representative of 3 independent experiments. (**B**) Neutrophils from people in the control group (*n* = 5) were treated with PBS, 2-DG (10 mM), G6PDi-1 (50 μM), or DPI (10 μM) for 30 minutes and stimulated with total IgG fractions prepared from people in the control group (control IgG, 10 μg/mL), total IgG fractions prepared from patients with APS (APS IgG, 10 μg/mL), PMA (40 nM), or Ca iono (10 μM) for 3 hours and NETosis was quantified using SYTOX Green. All data are presented as fold change compared with neutrophils that were not treated with any inhibitors or stimuli. (**C**) Neutrophils from people in the control group (*n* = 3) were treated with inhibitors as in **B** and then stimulated as indicated for 1 hour. Cytosolic ROS production was quantified using the Amplex Red reagent. For **B**–**C**, # represents the effectiveness of NETosis induction or ROS production for APS IgG, PMA, and Ca iono compared with control IgG using 1-way ANOVA with Holm-Šidák’s multiple comparison test; ^#^*P* < 0.05, ^####^*P* < 0.0001 * represents the change in NETosis or ROS production with the inhibitors in each stimulant group using 1-way ANOVA with Holm-Šidák’s multiple comparison test; **P < 0.01, ***P < 0.001, ****P < 0.0001.

We were next interested in identifying the metabolic determinants of APS IgG-induced NETosis (18, 22, 23). We also sought to delineate whether ROS formation for APS IgG–induced NETosis was primarily derived from the PPP (the PPP generates NADPH, which is then used by NOX to produce cytosolic ROS) or mitochondria; as such, we included PMA (which triggers cytosolic ROS) and the calcium ionophore A23187 (Ca iono, which promotes mitochondrial ROS) as controls (34, 35). To determine the time course of metabolic changes induced by the different stimuli, we measured the extracellular acidification rate (ECAR) and the oxygen consumption rate (OCR) over 4 hours using a metabolic flux analyzer (Figure 2A). With this experimental setup, the OCR is likely to primarily measure the massive oxygen consumption required for neutrophils to generate cytosolic ROS (39). As expected, we found that control IgG did not cause any significant changes in ECAR or OCR, while PMA-induced ECAR and OCR both peaked approximately an hour after stimulation. In contrast, Ca iono induced sharp ECAR and OCR changes within minutes of stimulation. APS IgG showed a unique pattern with a slow increase in ECAR and OCR that persisted over several hours. This time course aligns well with the timing of APS IgG-associated NETosis, which starts to increase around 90 minutes after stimulation (Supplemental Figure 1A).

Next, we found that APS IgG, PMA, and Ca iono induced more NETosis than did control IgG (Figure 2B). However, only APS IgG- and PMA-induced NETosis were sensitive to inhibition by 2-DG, G6PDi-1 (a recently described small molecule G6PD inhibitor) (45), or diphenyleneiodonium chloride (DPI, an inhibitor of NOX), results that together suggest roles for both glycolysis and the PPP (Figure 2B). Notably, 2-DG inhibits hexokinase and thus blocks flux through both glycolysis and the PPP (46), and G6PDi-1 specifically inhibits G6PD (45). For Ca iono–induced NETosis, there was no reduction with 2-DG, G6PDi-1, or DPI. We then measured cytosolic ROS formation and found that APS IgG and PMA, but not Ca iono, induced more ROS production than control IgG (Figure 2C). As with NETosis, the cytosolic ROS production triggered by APS IgG and PMA was drastically reduced in the presence of 2-DG, G6PDi-1, or DPI (Figure 2C). In a different set of experiments, we found that affinity-purified anti-β_2_GPI IgG induced NETosis and cytosolic ROS production to a similar degree as APS IgG (Supplemental Figure 1, B and C).

Figure 1B shows a possible intermediate phenotype for the small number of aPL-only patients enrolled in the study. To explore this further and to determine their potential effects on ROS production and metabolic flux, we cultured control neutrophils with individual IgG fractions isolated from the 9 patients with aPL-only (Figure 1B). Neutrophils stimulated with IgG fractions from 2 of the 9 patients with aPL-only (patients 7 and 8) triggered small increases in ROS production compared with control IgG (Supplemental Figure 1D). However, we did not see changes in neutrophil ECAR or OCR with any of the aPL-only IgG fractions (Supplemental Figure 1E). Interestingly, the 2 IgG fractions that triggered ROS production were from the only 2 patients in this group with detectable anti-β_2_GPI IgG activity.

**Figure 3:**
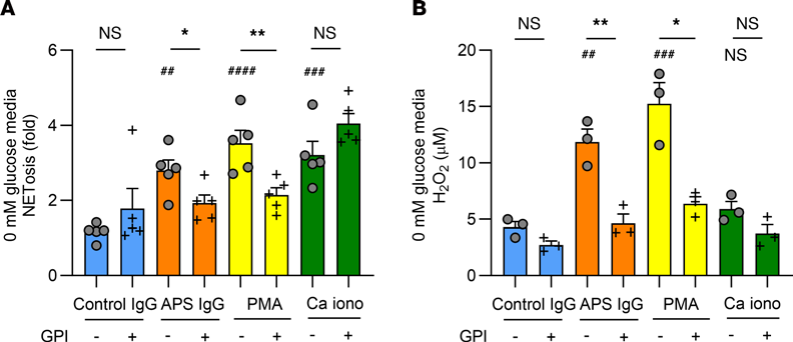
Glycogenolysis is necessary for APS IgG–induced human neutrophil NETosis and ROS production when glucose is absent from the culture media. These experiments were conducted in media without any glucose. (**A**) Neutrophils from controls (*n* = 5) were treated with PBS or GPI (10 μM) for 30 minutes and stimulated with control IgG (10 μg/mL), APS IgG (10 μg/mL), PMA (40 nM), or Ca iono (10 μM) for 3 hours. NETosis was quantified using SYTOX Green. All data are presented as fold change compared with neutrophils that were not treated with any inhibitors or stimuli. (**B**) Neutrophils from controls (*n* = 3) were treated with inhibitors as in **A** and then stimulated as indicated for 1 hour and cytosolic ROS production was quantified using the Amplex Red reagent. For **A** and **B**, # represents the effectiveness of NETosis induction or ROS production for APS IgG, PMA, and Ca iono compared with control IgG using 1-way ANOVA with Holm-Šidák’s multiple comparison test; ^##^*P* < 0.01, ^###^*P* < 0.001, ^####^*P* < 0.0001. * represents the change in NETosis or ROS production with the inhibitors in each stimulant group using *t* test; *P < 0.05, **P < 0.01.

We also evaluated the effect of a glycogen phosphorylase inhibitor (GPI), which acts to block glycogenolysis (47), on NETosis and cytosolic ROS production. In glucose-containing media, GPI did not show any effect on NETosis or ROS production (data not shown). However, when neutrophils were cultured in glucose-free media, GPI reduced NETosis and ROS production for APS IgG and PMA, but not Ca iono (Figure 3).

**Figure 4:**
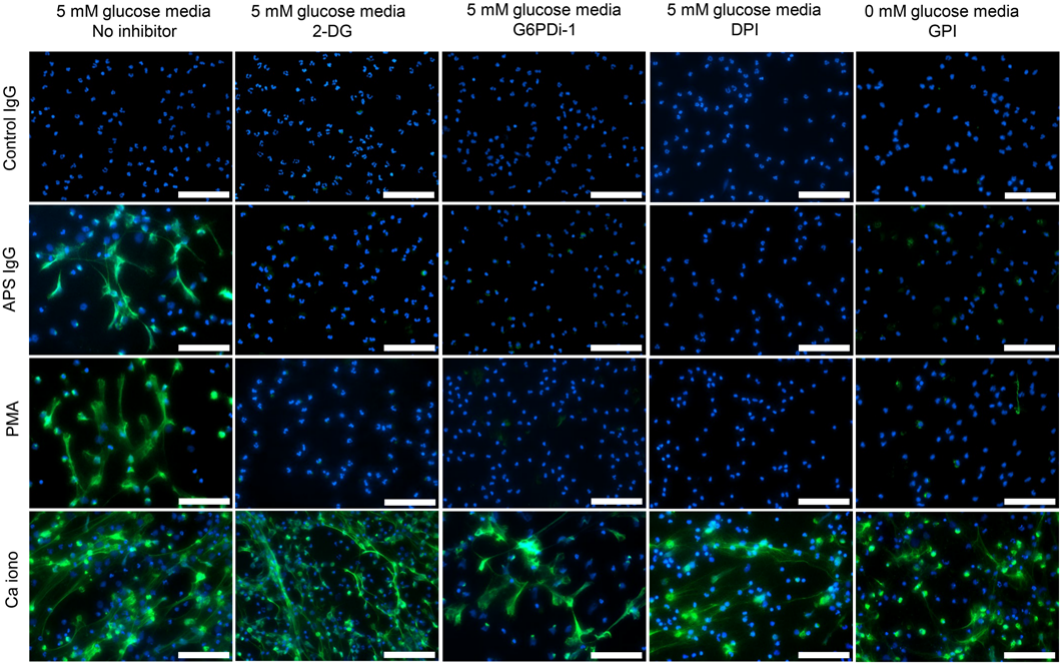
Representative immunofluorescence microscopy for stimulants and inhibitors as indicated. Blue,DNA; green, neutrophil elastase; scale bars: 100 μm. Representative of 3 independent experiments.

The plate-based NETosis data were confirmed by immunofluorescence microscopy with costaining for DNA and neutrophil elastase (Figure 4). Collectively, these data demonstrate that APS IgG–induced NETosis requires glycolysis and the PPP, with APS-IgG–induced neutrophil activation occurring along a slower time course compared with stimuli like PMA.

### APS IgG promotes metabolic changes in neutrophils.

We observed that in the presence of APS IgG neutrophils released more lactate, which could be reversed in the presence of 2-DG, but not G6PDi-1 or DPI (Supplemental Figure 2A). We also observed a numerical but not a statistically significant increase in the enzymatic activity of G6PD with APS IgG, which was reduced by G6PDi-1 but remained stable with 2-DG and DPI (Supplemental Figure 2B). Using flow cytometry, we confirmed that neutrophils increase their total ROS production when stimulated with APS IgG (Supplemental Figure 2C). Moreover, treatment with any of 2-DG, G6PDi-1, or DPI reduced ROS production to levels seen with control IgG-stimulated neutrophils (Supplemental Figure 2C). We did not observe any differences in the above parameters when GPI was used (data not shown).

We next questioned whether APS IgG promotes neutrophil glucose 6-phosphate (G6P) and NAPDH accumulation. An hour after stimulation, we observed that G6P increased to approximately 2-fold in neutrophils stimulated with APS IgG before returning to baseline (Supplemental Figure 2D). APS IgG-stimulated neutrophils produced more NADPH for 2 hours before decreasing to the levels seen in control IgG-stimulated neutrophils (Supplemental Figure 2E). We also observed an increase in intracellular glycogen with APS IgG when neutrophils were incubated in glucose-containing media but not in glucose-free media (Supplemental Figure 2F). These data suggest that APS IgG can increase lactate, G6P, NADPH, and total ROS production by neutrophils.

### In mice, 2-DG restrains APS IgG–associated NETosis without impacting bleeding time.

**Figure 5:**
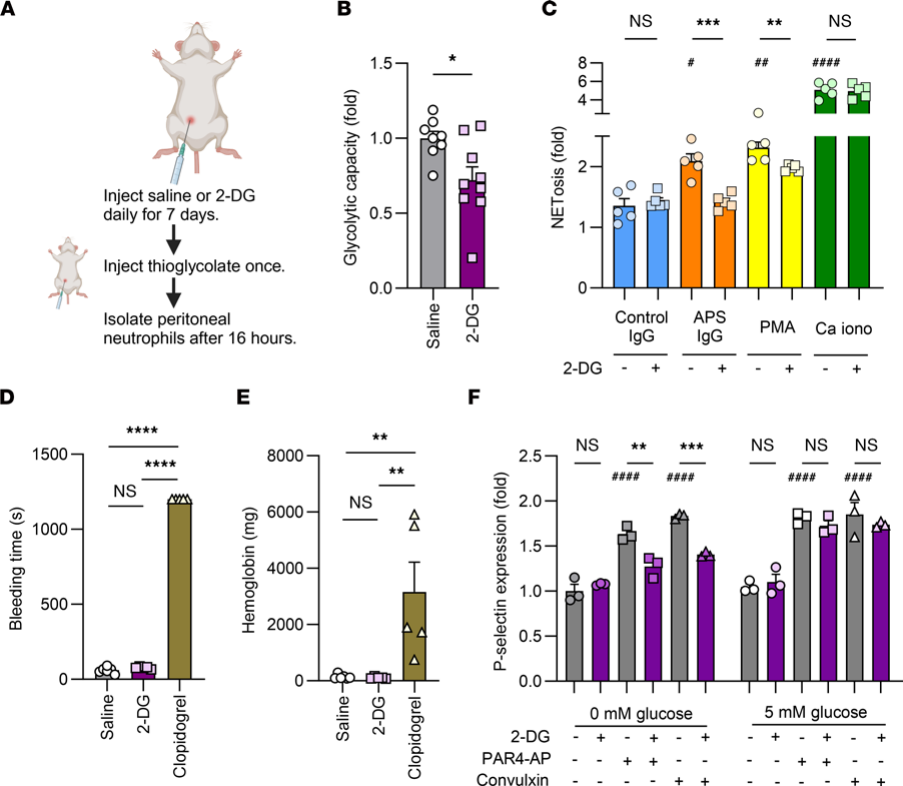
In mice, 2-DG restrains neutrophil glycolysis, ROS production, and NETosis, but does not impact bleeding time. (**A**) Timeline of treatment with saline or 2-DG (0.5 g/kg) and peritoneal neutrophil isolation. (**B**) Glycolytic capacity was measured and presented as fold change in the 2-DG–treated mice (*n* = 9) compared with the saline-treated mice (*n* = 8). *P < 0.05 using *t* test. (**C**) Neutrophils from mice administered saline or 2-DG (*n* = 5) were stimulated with control IgG (10 μg/mL), APS IgG (10 μg/mL), PMA (250 nM), or Ca iono (10 μM,) and NETosis was quantified using SYTOX Green. Data are presented as fold change compared with neutrophils from saline-treated mice that were left unstimulated. # represents the effectiveness of NETosis induction as compared with control IgG using 1-way ANOVA with Holm-Šidák’s multiple comparison test; ^#^*P* < 0.05, ^##^*P* < 0.01, and ^####^*P* < 0.0001. * represents the change in NETosis in neutrophils from mice administered 2-DG compared with the mice administered saline in each stimulant group; **P < 0.01, ***P < 0.001 using *t* test. (**D** and **E**) C57BL/6J mice were treated with saline (*n* = 6), 2-DG (*n* = 5, 0.5 g/kg), or clopidogrel (*n* = 5, 2.5 mg/kg) daily for 7 days followed by quantification of (**D**) tail vein bleeding time and (**E**) accumulated hemoglobin. **P < 0.01, ****P < 0.0001 using 1-way ANOVA with Holm-Šidák’s multiple comparison test. (**F**) Washed platelets from C57BL/6J mice (*n* = 3 mice) were isolated and cultured in 0 or 5 mM glucose-containing buffers and then treated with PBS or 2-DG (10 mM) for 30 minutes followed by treatment with platelet agonists as indicated. Platelet P-selectin expression was quantified. # represents the change in P-selectin expression in platelets treated with PAR4-AP and convulxin compared with untreated platelets and * represents the change in P-selectin expression with 2-DG in each platelet agonist group using 1-way ANOVA with Holm-Šidák’s multiple comparison test; ^**^P < 0.01, ^***^P < 0.001, ^####^*P* < 0.0001.

We sought to determine whether administering 2-DG to mice would replicate the data seen above in human neutrophils. To accomplish this, we administered intraperitoneal saline or 2-DG to C57BL/6J mice once daily for 7 days. This was followed by induction of thioglycolate-induced peritonitis and isolation of neutrophils after 16 hours (Figure 5A). After isolation, neutrophils were not exposed to any additional 2-DG.

We evaluated the glycolytic capacity of isolated peritoneal neutrophils and found that 2-DG treatment reduced their ability to use glycolysis (Figure 5B). We also found that 2-DG administration decreased NETosis induced by APS IgG and PMA, but not by Ca iono (Figure 5C). Next, we evaluated bleeding times as a blunt approximation of platelet function. Bleeding was not significantly different between the saline- and 2-DG–treated mice but was markedly increased in mice given the antiplatelet agent clopidogrel as a positive control (Figure 5D). We found comparable results when quantifying the accumulated hemoglobin content after tail vein bleeding (Figure 5E). There were no changes in body weight, circulating blood glucose, total white blood cell count, absolute neutrophil count, or hemoglobin between the saline- and 2-DG-treated mice (data not shown); however, 2-DG administration did modestly reduce platelet counts (1,402,000/μl ± 91,000 with saline versus 962,000/μl ± 101,000 with 2-DG; *P* < 0.0001 by *t* test). Finally, to provide a more nuanced assessment of platelet function, we quantified whether platelet P-selectin expression was affected by ex vivo treatment with 2-DG. When platelets were isolated and cultured in glucose-containing buffers, 2-DG did not affect P-selectin expression; however, in glucose-free buffers, 2-DG did effectively reduce P-selectin expression in response to stimulation with either a PAR4 agonist peptide or the GPVI agonist convulxin (Figure 5F).

Taken together, these results suggest that administering 2-DG to mice reduces the ability of peritoneal neutrophils to utilize glycolysis and release NETs. Further, 2-DG reduces P-selectin expression in a glucose-free buffer, suggesting potential impacts on platelet function that do not extend to bleeding time.

### When administered to mice, APS IgG promotes 2-NBDG uptake, glycolysis, and NETosis.

**Figure 6:**
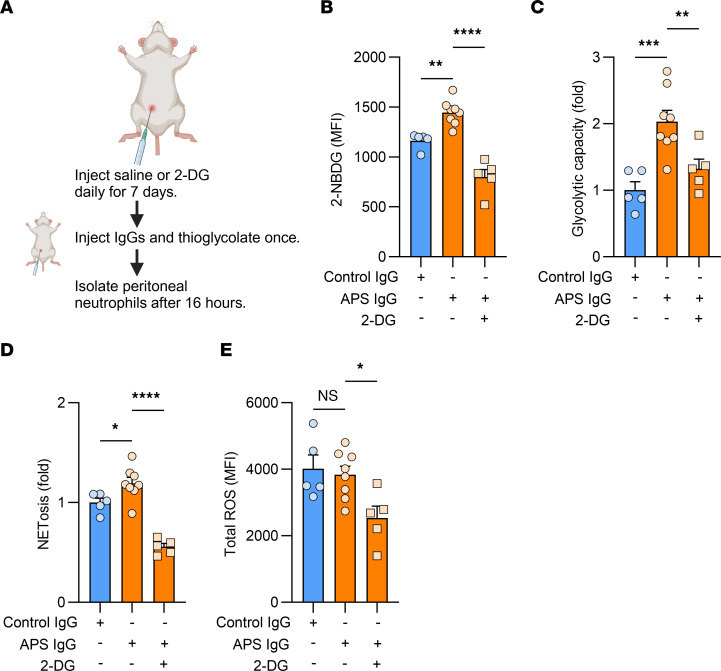
In mouse neutrophils, APS IgG promotes NETosis, 2-NBDG uptake, and glycolysis that can be restrained by 2-DG. (**A**) Timeline of treatment with saline or 2-DG (0.5 g/kg), intraperitoneal IgG administration, and peritoneal neutrophil isolation. Each point represents 1 mouse; *n* = 5 for control IgG + saline, *n* = 8 for APS IgG + saline, and *n* = 5 for APS IgG + 2-DG. (**B**) 2-NBDG fluorescence was measured using flow cytometry. (**C**) Glycolytic capacity was measured using the glycolysis stress test. (**D**) Spontaneous NETosis was characterized using SYTOX Green. For **C** and **D**, data are presented as fold change in the APS IgG-treated mice compared with the control IgG-treated mice. (**E**) Total cellular ROS production was measured with DCFDA fluorescence. For all, *P < 0.05, **P < 0.01, ***P < 0.001, ****P < 0.0001 using 1-way ANOVA with Holm-Šidák’s multiple comparison test.

Next, we aimed to identify whether directly administering APS IgG to mice changes neutrophil metabolism, as well as the extent to which this can be influenced by metabolic inhibitors. To do this, we treated C57BL/6J mice with once-daily itraperitoneal saline or 2-DG for 7 days. At the end of the 7 days, we administered a 1-time dose of 2 mg control or APS IgG into the peritoneal cavity along with thioglycolate to trigger neutrophil recruitment. Peritoneal neutrophils were isolated after 16 hours (Figure 6A). After isolation, the neutrophils were not treated with additional IgGs or 2-DG.

Using flow cytometry, we observed that 2-NBDG fluorescence was increased in the neutrophils of mice administered APS IgG and that 2-DG was able to reverse this (Figure 6B). We found the same relationship for glycolytic capacity (Figure 6C) and spontaneous NETosis (Figure 6D). We did not observe significant changes in total ROS production with APS IgG compared with control IgG, even though ROS production decreased with 2-DG (Figure 6E), possibly suggesting that peritonitis activated the neutrophils and diminished the additive effects of APS IgG on ROS production.

To a different set of mice, we administered saline or G6PDi-1 daily for 3 days, after which we injected 2 mg of APS IgG to each mouse along with thioglycolate and isolated peritoneal neutrophils after 16 hours (Supplemental Figure 3A). G6PDi-1 was able to reduce spontaneous NETosis and total ROS production (Supplemental Figure 3, B and C). In summary, APS IgG administration to mice increased glycolytic capacity and NETosis in peritoneal neutrophils, which could be decreased by 2-DG.

### APS IgG–induced venous thrombosis in mice is dependent on glycolysis and the PPP.

**Figure 7:**
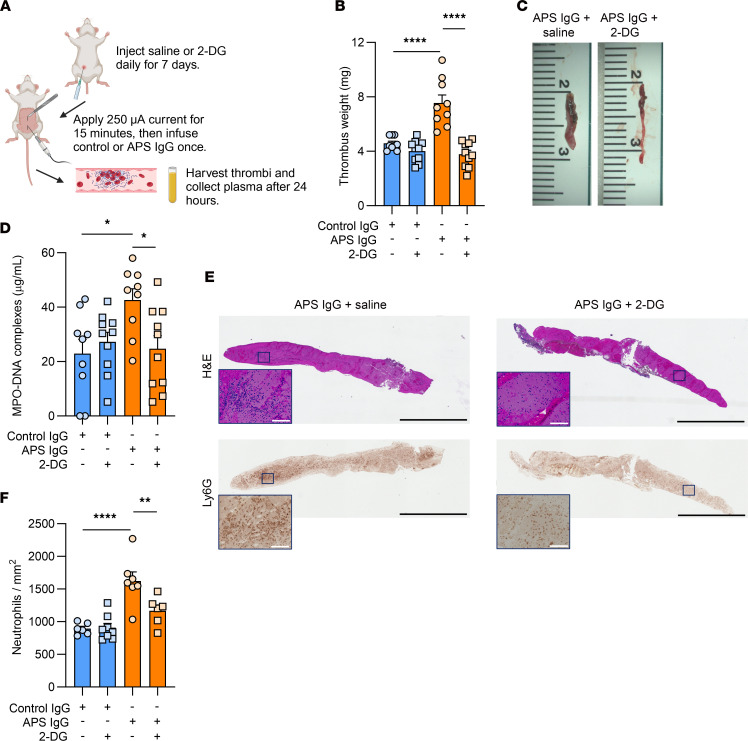
2-DG mitigates APS IgG-induced thrombosis in mice. (**A**) Time of treatment with saline or 2-DG (0.5 g/kg) and schematic of APS IgG-induced electrolytic injury model. (**B**) Thrombus weights; each point represents 1 mouse, *n* = 9 for each saline-treated group and *n* = 10 for each 2-DG-treated group. ****P < 0.0001 using 1-way ANOVA with Holm-Šidák’s multiple comparison test. (**C**) Representative thrombi from mice treated with APS IgG and saline (left) or APS IgG and 2-DG (right). (**D**) Plasma MPO-DNA complexes as a measure of circulating NETs; each point represents 1 mouse; *P < 0.05 using 1-way ANOVA with Holm-Šidák’s multiple comparison test. (**E**) Representative H&E- and Ly6G-stained thrombus sections from mice treated with APS IgG and saline (left) or APS IgG and 2-DG (right); full thrombus is at ×4 magnification, scale bars: 2,000 μm; inset is ×20 magnification, scale bars: 100 μm. (**F**) Quantification of neutrophil infiltration in Ly6G-stained thrombus sections. Each point represents a section from 1 mouse, *n* = 6 for control IgG + saline, *n* = 8 for control IgG + 2-DG, *n* = 7 for APS IgG + saline, and *n* = 6 for APS IgG + 2-DG; *P < 0.05, **P < 0.01, ****P < 0.0001 using 1-way ANOVA with Holm-Šidák’s multiple comparison test.

Lastly, we wanted to determine whether glycolysis or the PPP are necessary for APS-associated thrombosis. We used the electrolytic inferior vena cava (IVC) injury model of venous thrombosis that we have described previously (20, 21, 48); mice treated with IgG from patients with APS form larger thrombi compared with mice treated with IgG from controls (Figure 7A). As expected, we found that APS IgG promoted larger thrombi than control IgG (Figure 7B). In the APS mice that also received 2-DG, there was a marked reduction in thrombus size, comparable to mice that had received control IgG (Figure 7, B and C). This decrease in thrombus burden was accompanied by a reduction in circulating NET remnants, measured using an ELISA quantifying MPO-DNA complexes (Figure 7D). Figure 7E shows representative histology of thrombus sections stained with H&E, as well as Ly6G as a marker of neutrophils. We quantified the number of thrombus-infiltrating Ly6G-positive neutrophils, which were significantly reduced in the mice treated with 2-DG (Figure 7F). When mice were given G6PDi-1, we again found a reduction in APS-associated thrombus weights and circulating MPO-DNA complexes (Supplemental Figure 3, D–F). In summary, glycolysis and PPP inhibition can reduce APS IgG–induced venous thrombosis in mice.

## Discussion

Historically, APS has primarily been thought of as a thrombotic disease; however, there are also clearly inflammatory aspects to APS with innate leukocytes and proinflammatory mediators implicated in its pathogenesis (11, 23, 49). Despite this, the cornerstone of the treatment paradigm in APS remains antithrombotic agents like warfarin, rather than immunomodulation (50). Although anticoagulation appears to have benefits for patients with purely thrombotic complications, it is estimated that up to 20% of these patients will still go on to have recurrent thrombosis (4). Anticoagulation is even less effective in patients with APS with microvascular disease manifestations, which bring with them a high degree of morbidity and mortality (5, 6). In addition, newer antithrombotic agents that are easier to manage than warfarin may be less effective in APS due to an increased risk of arterial thrombotic events (6). Antithrombotic agents also obviously predispose patients to bleeding complications. For all of these reasons, there is an ongoing need to understand the inflammatory pathophysiology of APS in the pursuit of developing better and more targeted therapies.

Neutrophils and NETs are implicated in the pathogenesis of APS and modulating their hyperactivity is an effective strategy to reduce thrombotic events in mice (23). In addition, by RNA-Seq of patient neutrophils, we have found that genes involved in glycolysis, glycogenolysis, and the PPP were upregulated in APS neutrophils. These 3 pathways are particularly relevant for neutrophils, which have fewer mitochondria than other cells (51–53). Glycogen provides glucose for glycolysis in nutrient-limited contexts and can be modified in inflammatory environments (54–58). The PPP is critical in fueling the oxidative burst that is a major aspect of neutrophil function (29–33). Of note, both the glycogen pathway and the PPP branch off of the upper glycolytic intermediate, G6P, which is converted from glucose by hexokinase. Here, we focused on these metabolic pathways as they seem to be particularly relevant for neutrophils.

To further understand possible metabolic changes in APS neutrophils, we profiled neutrophils from our well-characterized cohort of patients with APS using metabolic flux analysis. Compared with controls or patients with non-APS thrombosis, patients with APS had neutrophils that engaged glycolysis more avidly (*P* < 0.05 for both comparisons). However, there was not a statistically significant difference when APS neutrophils were compared with neutrophils isolated from a relatively small number of patients with circulating aPL antibodies but no clinical manifestations of APS; neutrophils from the aPL-only patients appeared to display an intermediate phenotype. Interestingly, when we purified total IgG fractions from the patients with aPL-only and tested each fraction individually, 2 fractions did trigger modest ROS production by neutrophils; these were notably the only 2 fractions with anti-β_2_GPI IgG activity. None of the aPL-only IgG fractions affected neutrophil ECAR or OCR. Taken together, these results suggest that the aPL-only group may tend to demonstrate less neutrophil activity because they are less likely to have triple-positive aPL antibody profiles. It is known that 1%–5% of patients with circulating aPL antibodies will go on to develop APS manifestations (6); how to appropriately risk stratify and manage patients with aPL-only is an ongoing question in the field. We hope to evaluate the potential contribution of neutrophil metabolism to this risk with future studies in a larger number of patients with aPL-only, including some with triple-positive aPL antibody profiles. Another point to note is that we profiled normal-density neutrophils in this study, and it will be of interest in future studies to also characterize low-density neutrophils. There are conflicting reports about the number of low-density neutrophils in APS; however, when present, they are likely to be less mature, and it would be interesting to determine whether they are metabolically different, as has been described for a cancer model (18, 59–62).

Intriguingly, neutrophils from patients with APS with microvascular complications tended to have the highest glycolytic capacities. This increase in their ability to engage glycolysis raises the question of whether targeting glycolytic metabolism might be a novel approach to treatment. It has previously been shown that extracellular glucose may be limited at sites of inflammation (58, 63); however, when glucose is abundant, neutrophils can increase their stores of glycogen (64–66). This is especially true for activated neutrophils, which can be found in the settings of APS, ongoing COVID-19 infection, or when healthy neutrophils are stimulated with PMA, zymosan, TNF-α, or APS IgG (64–66).

When glycogenolysis is inhibited and glucose is absent from the culture media (mimicking some inflammatory tissues), others have found reduced PMA-induced NETosis and increased neutrophil apoptosis (64, 66). Our experiments found something similar. In media replete with glucose, inhibiting glycogenolysis did not affect APS-associated NETosis. This is presumably because neutrophils can easily use extracellular glucose to support NETosis. However, when glucose was absent, inhibiting glycogenolysis profoundly reduced APS-associated NETosis. We believe that these findings demonstrate the importance of glycolysis for supporting APS-associated NETosis and that neutrophils can be metabolically flexible and use glycogen-derived glucose to fuel glycolysis when needed.

While there have been reports assessing the metabolic requirements of neutrophils when confronted with nonphysiologic and microbial stimuli (36–39), their response to physiologic stimuli that are relevant to autoimmune diseases are understudied. Here, we determined that APS IgG can induce metabolic changes, especially in terms of oxygen consumption, but in a pattern that is distinct from that seen with PMA and the Ca iono. When stimulated with APS IgG, neutrophils had a slower onset of oxygen consumption but could sustain it over a longer period of time. We have also demonstrated that neutrophils stimulated with APS IgG accumulate G6P, which likely then fuels G6PD-induced NADPH production. This NADPH can then be used by NOX to produce ROS. Interrupting this sequence of events at any point reduces APS IgG–mediated ROS production and NETosis.

Rabbits with venous thrombi have higher levels of circulating lactate, suggesting that thrombosis is a glycolysis-inducing process in mammals (67); the experimental setup in this model used endothelial denudation and vessel ligation, causing venous stasis (67). In humans with acute coronary syndromes, the presence of hyperglycemia (even in the absence of diabetes mellitus) has been associated with increased local thrombin generation and longer clot lysis times (68). Here, we wanted to evaluate whether glycolysis (2-DG) and the PPP (G6PDi-1) were relevant for APS-associated venous thrombosis. We found that administering 2-DG in the context of an APS IgG–induced venous thrombosis model led to reductions in thrombus size, neutrophil infiltration into thrombi, and circulating NET markers. We also found that 2-DG reduced P-selectin expression when platelets were isolated and cultured in buffers without glucose; however, in contrast to the neutrophil experiments, this effect was not seen when glucose-containing buffers were used. Though 2-DG did not impact bleeding times (a relatively blunt measure of platelet function) in mice, it is certainly possible that some platelet functions were affected in vivo. This important possibility should be explored in future APS studies, especially in arterial vascular beds where platelets surely play an important role in APS-associated thrombosis. Taken together, our study builds upon existing literature about the role of metabolism in thrombosis (69–71), further reinforcing that metabolic modulation can affect thrombosis in a human disease model in a manner that seems at least partially dependent on neutrophils.

An outstanding question is whether the decreased neutrophil infiltration in thrombus sections is due to decreased chemotaxis. However, others have demonstrated a role for mitochondrial metabolism more so than glycolysis for chemotaxis. For example, in response to chemotactic stimuli, mitochondrial metabolism and mTOR signaling promote chemotaxis while purinergic signaling inhibits it (72). Another group has shown that inhibiting mitochondrial function by using either oligomycin (an ATP synthase inhibitor) or FCCP (a depolarizing agent) inhibited chemotaxis (27). Similarly, metformin (a complex I inhibitor) has been shown to inhibit neutrophil chemotaxis (73). In zebrafish, neutrophil-specific deletion of mitochondrial DNA polymerase reduced neutrophil motility (74). Finally, neutrophils with deficient isocitrate dehydrogenase, a key enzyme in the TCA cycle, also have impaired chemotaxis (75). Based on our findings, the extent to which glycolysis inhibition also affects neutrophil chemotaxis deserves characterization in future studies.

Our study has limitations. The patient characterizations were performed only in a cross-sectional fashion, and futures studies are planned to longitudinally profile patient subsets to determine whether changes in neutrophil metabolism can predict future clinical events. The observational nature of this study also makes it difficult to identify the primary drivers of the demonstrated metabolic changes. In addition, gene knockdown experiments are not possible in primary neutrophils, thereby making it more challenging to identify the specific metabolic enzymes that directly contribute to the disrupted physiology, which is a topic that we will attempt to tackle over time.

In conclusion, this study shows the promise of leveraging metabolism as a treatment approach in APS, as has been considered recently for other autoimmune diseases (76–78). Our study demonstrates that neutrophil glycolysis is enhanced in patients with APS and that subsets of these patients can be defined based on their metabolism. Further, we have shown that inhibiting glycolysis can protect against thrombosis. Future studies will focus on identifying the effect of glycolysis on other cells important in thrombosis, such as platelets and endothelial cells, and how to sustainably modulate glycolysis utilization in patients. Such approaches could have broad impacts in disease states beyond APS.

## Methods

### Sex as a biological variable

Our study examined both male and female humans. Mouse studies were limited to male mice due to the risk of ovarian necrosis with the IVC thrombosis model (79).

### Human participants

Patients with APS (*n* = 34) (1) and patients with circulating aPL antibodies but no criteria defining APS manifestations (aPL-only, *n* = 9) were recruited from the Rheumatology clinic at the University of Michigan for fresh blood donation. None of the patients met the American College of Rheumatology criteria for systemic lupus erythematosus (80). Patients with thrombosis without aPL antibodies [Thromb (aPL–), *n* = 9] were recruited from hematology clinics at the University of Michigan. Healthy participants (*n* = 42) were recruited through an online posting. Exclusion criteria included a history of systemic autoimmune disease, active infection, and pregnancy. Blood was collected by phlebotomist venipuncture.

### Purification of IgG

IgG was purified from APS, aPL-only, or control sera with a Protein G Agarose Kit (Pierce) following the manufacturer’s instructions, as previously described (18, 20). All IgG samples were free of detectable endotoxin as determined by the Pierce LAL Chromogenic Endotoxin Quantitation Kit (88282) according to the manufacturer’s instructions.

### Quantification of aPL antibodies

aPL antibodies were quantified from plasma using Quanta Lite assay kits from Inova Diagnostics (anti-β_2_GPI IgG, 708665; anti-β_2_GPI IgM, 708670; anti-PS/PT IgG, 708835; anti-PS/PT IgM, 708845) according to the manufacturer’s instructions, as previously described (81).

### Human neutrophil purification

Blood was collected into heparin tubes by standard phlebotomy techniques. The anticoagulated blood was then fractionated by density-gradient centrifugation using Ficoll-Paque Plus (GE Healthcare). Neutrophils were further purified by dextran sedimentation of the red blood cell layer before lysing residual red blood cells with 0.2% sodium chloride. Neutrophil preparations were at least 95% pure, as confirmed by flow cytometry and nuclear morphology.

### Flow cytometry assays relevant to multiple experiments with human neutrophils

For experiments assessing the metabolic parameters of patients (i.e., for Figure 1), neutrophils (1×10^6^) from people in the control group or patients with APS were used immediately after purification. For experiments assessing the metabolic changes promoted by APS IgG (i.e., for Supplemental Figure 2), purified neutrophils (1 × 10^6^) from controls were incubated for 30 minutes with phosphate-buffered saline (PBS), 10 mM 2-DG (Sigma-Aldrich), 50 μM G6PDi-1 (Sigma-Aldrich), 10 μM DPI (Sigma-Aldrich), or 10 μM GPI (Cayman Chemical). Then, they were stimulated with 10 μg/mL control IgG (pooled from 3 individuals) or 10 μg/mL APS IgG (pooled from 3 patients with APS who were triple positive for lupus anticoagulant testing, anti-β_2_GPI IgG, and anticardiolipin IgG) for 2 hours in SILAC RPMI 1640 FLEX media (Gibco) supplemented with 2 mM glutamine (Gibco), 1% heat-inactivated FBS (Gibco), with or without 5 mM glucose (Sigma-Aldrich), and used in flow cytometry experiments. For all experiments, prior to staining, Fc blocking of cells was carried out using TruStain FcX (BioLegend, 422302) according to the manufacturer’s instructions, and a BioRad-ZE5 flow cytometer was used. Analysis was done with FlowJo software (Tree Star).

#### 2-NBDG assay.

Neutrophils were incubated with 200 μg/mL 2-NBDG (Invitrogen, N13195) for 30 minutes at 37°C. Cells were then washed,and fluorescence was measured.

#### Total cellular ROS quantification.

Neutrophils were stained with 0.5 μM H2DCFDA (Invitrogen, C6827) for total ROS. Neutrophils were marked with APC-conjugated CD10 (BioLegend, 312210), PE-conjugated CD16 (BD Pharmingen, 555407), or pacific blue-CD15 (BioLegend, 394704), and viable cells were gated by Ghost dye violet 510 (Tonbo Biosciences, TB-13-0870-T100). After 30 minutes of incubation with fluorophores, cells were washed to remove excess dye and fluorescence was measured.

#### GLUT-1 quantification.

Neutrophils were stained with a 1:50 dilution of GLUT1 antibody (NOVUS Biologicals, NBP2-75786AF750) for 20 minutes at room temperature, fixed with 4% paraformaldehyde, and washed. Fluorescence was then measured.

### Seahorse extracellular flux assay

Extracellular flux analysis was conducted at 37°C without CO_2_ using the XFe96 Seahorse Extracellular Flux Analyzer (Agilent). For all metabolic flux experiments, XF RPMI, media supplements, and stress test reagents were purchased from Agilent. Purified neutrophils (both human and mouse) were plated on 0.001% poly L-lysine-coated (Sigma-Aldrich) Seahorse cell culture plates at a concentration of 2×10^5^/well in XF RPMI. For the glycolysis stress test, XF RPMI was supplemented with 2 mM glutamine, then serially treated with 10 mM glucose, 2 μM oligomycin, and 50 mM 2-DG according to the standard stress test protocol provided by Agilent. For some experiments, neutrophils from controls were treated with control IgG (10 μg/mL), APS IgG (10 μg/mL), aPL-only IgG (10 μg/mL), PMA (40 nM, Sigma), or Ca iono (10 μM, Cayman Chemical), and changes in ECAR and OCR were measured over 4 hours. These experiments were conducted in XF RPMI supplemented with 2 mM glutamine, 10 mM glucose, and 1 mM pyruvate.

### Quantification of lactate release

Purified neutrophils (1×10^6^) from people in the control group or patients with APS were added to 24-well tissue culture plates (Thermo Fisher Scientific) and incubated for 2 hours in SILAC RPMI 1640 FLEX media supplemented with 2 mM glutamine, 1% heat-inactivated FBS, and 5 mM glucose. The plate was centrifuged and the media supernatant was collected, snap frozen in liquid nitrogen, and stored at –80°C. For some experiments, as was done for the flow cytometry experiments above, purified neutrophils (1×10^6^) from controls were treated with inhibitors for 30 minutes, then stimulated with IgGs for 2 hours. The media supernatant was collected and stored as described. The released lactate was quantified using an assay kit (Abcam, ab65330) according to the manufacturer’s instructions. Absorbance was measured at 570 nm using a Cytation 5 Cell Imaging Multi-Mode Reader (BioTek).

### Quantification of G6PD enzyme activity

Purified neutrophils (1×10^6^) from controls or patients with APS were added to 24-well tissue culture plates and incubated for 2 hours in SILAC RPMI 1640 FLEX media supplemented with 2 mM glutamine, 1% heat-inactivated FBS, and 5 mM glucose. The plate was centrifuged, the cells were washed with PBS, the wells were scraped, and the cells was collected in 100 μL ice-cold PBS. A lysate was generated by sonicating on ice for 5 seconds. After centrifuging at 4°C to remove debris, the supernatant was collected, snap frozen in liquid nitrogen, and stored at –80°C. For some experiments, purified neutrophils (1×10^6^) from controls were treated with inhibitors and IgGs as for the earlier experiments, and the cell lysate supernatant was collected and stored as described. The G6PD enzyme activity was quantified using an assay kit (Abcam, ab176722) according to the manufacturer’s instructions. Fluorescence was measured at 540/590 nm excitation/emission using a Cytation 5 Cell Imaging Multi-Mode Reader.

### Quantification of intracellular glycogen

Purified neutrophils from people in the control group or patients with APS (0.25×10^6^) were collected, lysed with 100 μL sterile water, boiled to neutralize enzymes, and stored at –80°C. For some experiments, purified neutrophils (0.25×10^6^) from people in the control group were treated with control or APS IgG and incubated for 2 hours in SILAC RPMI 1640 FLEX media supplemented with 2 mM glutamine, 1% heat-inactivated FBS, and either 0 or 5 mM glucose media, and the cell lysate was collected and stored as described. The glycogen content was measured with the Glycogen assay kit (Abcam, ab65620) according to the manufacturer’s instructions. Fluorescence was measured at 535/587 nm excitation/emission using a Cytation 5 Cell Imaging Multi-Mode Reader.

### NETosis assays

To assess NETosis, neutrophils (1×10^5^/well) from controls were cultured in 96-well black-wall clear-bottom tissue culture plates (Thermo Fisher Scientific) in SILAC RPMI 1640 FLEX media supplemented with 2 mM glutamine, 1% heat-inactivated FBS, 250 nM Sytox Green (Invitrogen), and with or without 5 mM glucose. They were incubated for 30 minutes with PBS, 10 mM 2-DG, 50 μM G6PDi-1, 10 μM DPI, or 10 μM GPI and then stimulated with PBS, 10 μg/mL control IgG, 10 μg/mL APS IgG, 10 μg/mL affinity-purified anti-β_2_GPI IgG, 40 nM PMA, or 10 μM Ca iono for 3 hours. Fluorescence was measured at 504/523 (excitation/emission) using a Cytation 5 Cell Imaging Multi-Mode Reader. Data were normalized against the fluorescence obtained from neutrophils treated with PBS (i.e., no inhibitor and no stimulation). Each experiment was repeated with neutrophils isolated from 5 independent, healthy volunteers. For some experiments, neutrophils (1×10^5^/well) from controls were treated with PBS, 10 μg/mL control IgG, or 10 μg/mL APS IgG and fluorescence was measured every 20 minutes for 3 hours.

### Cytosolic ROS quantification assay

The generation of cytosolic ROS was quantified as described previously (17). Briefly, neutrophils (1×10^5^/well) from controls were treated with the above inhibitors for 30 minutes and then with the above stimuli for 60 minutes, and ROS production was detected by a colorimetric assay using 50 μM Amplex Red reagent (Invitrogen) and 10 U/mL horseradish peroxidase (Sigma-Aldrich) that was added to the culture medium. Absorbance was measured at 560 nm and linearity was assured with an H_2_O_2_ standard curve. Each experiment was repeated with neutrophils isolated from 3 independent healthy volunteers.

### Immunofluorescence microscopy

For immunofluorescence microscopy, 5×10^4^ neutrophils were seeded onto coverslips coated with 0.001% poly L-lysine (Sigma-Aldrich) and were treated with inhibitors and stimulants as above. Then, they were fixed with 4% paraformaldehyde for 15 minutes at room temperature and blocked overnight with 10% FBS in PBS at 4°C. The primary antibody was against neutrophil elastase (Sigma-Aldrich 481001, diluted 1:100), and the FITC-conjugated secondary antibody was from Southern Biotech (4052-02, diluted 1:250). DNA was stained with Hoechst 33342 (Invitrogen). Images were collected with a Cytation 5 Cell Imaging Multi-Mode Reader.

### Quantification of G6P and NADPH

Purified neutrophils (2×10^6^) from people in the control group were added to 24-well tissue culture plates, treated with 10 μg/mL control or APS IgG, and incubated for 0–3 hours in SILAC RPMI 1640 FLEX media supplemented with 2 mM glutamine, 1% heat-inactivated FBS, and 5 mM glucose. At each time point, the plate was centrifuged, the cells were washed with PBS, the wells were scraped, and the cells was collected in 100 μL ice-cold PBS. A lysate was generated by sonicating on ice for 5 seconds. After centrifuging at 4°C to remove debris, the supernatant was collected, snap frozen in liquid nitrogen, and stored at –80°C. G6P (Abcam, ab107923) and NADPH (Abcam, ab176724) were quantified according to the manufacturer’s instructions.

### Animal housing and treatments

Mice were housed in a specific pathogen-free barrier facility and fed standard chow. Experimental protocols were approved by the University of Michigan Institutional Animal Care and Use Committee, and all relevant ethical regulations were followed. Mice were purchased from The Jackson Laboratory. 10-week-old male C57BL/6J mice were administered 0.5 g/kg 2-DG (daily for 7 days, Sigma-Aldrich), 25 mg/kg G6PDi-1 (daily for 3 days, Sigma-Aldrich), or saline (daily for 3 or 7 days) by intraperitoneal injection. For some experiments, on the last day of the treatment with the agents above, control IgG (2 mg per mouse) or APS IgG (2 mg per mouse) was injected into the peritoneal cavity, and peritonitis was induced as described in the “mouse peritoneal neutrophil purification.

### Mouse peritoneal neutrophil purification

For some experiments, peritonitis was induced using a 3% Brewer thioglycolate medium (Thermo Fisher Scientific) according to a previous protocol (82), with some modifications. Thioglycolate was dissolved in distilled water, autoclaved, and aged for at least 2 months while avoiding light exposure. A total of 1 mL thioglycolate medium was administered to the mice intraperitoneally; for mice that were given intraperitoneal IgGs, thioglycolate was administered concurrently with the IgGs. After 16 hours, the mice were euthanized and 10 mL of ice-cold PBS with 2% FBS and 5 mM ethylenediaminetetraacetic acid was instilled into the peritoneal cavity, the abdomen was gently massaged, and the solution was lavaged. Peritoneal neutrophils were purified using the negative selection procedure from the EasySep mouse neutrophil enrichment kit (StemCell Technologies) per manufacturer’s instructions. We examined peritoneal neutrophils because bone marrow neutrophils have a more immature OXPHOS-utilizing phenotype (28).

### Mouse neutrophil extracellular flux assay

This was done using the technique described above for human neutrophils.

### Mouse NETosis assay

Neutrophils (1×10^5^/well) were isolated from 5 mice each (and kept separately) in the saline- and 2-DG-treated groups and then seeded in 96-well cell culture plates in the same media conditions as for the human NETosis assay above. They were then stimulated with PBS, 10 μg/mL APS IgG, 10 μg/mL control IgG, 250 nM PMA, or 10 μM Ca iono for 4 hours. Fluorescence was measured at 504/523 (excitation/emission) using a Cytation 5 Cell Imaging Multi-Mode Reader. Data were normalized against the fluorescence obtained from neutrophils from mice administered intra-peritoneal saline and stimulated with PBS ex vivo (i.e., no inhibitor and no stimulation). For some experiments, spontaneous NETosis without any additional stimuli was assessed. Importantly, the only metabolic inhibition that the cells were exposed to was via the intraperitoneal injections.

### Tail vein bleeding time

Mice were treated with intraperitoneal saline, 0.5 g/kg 2-DG, or 2.5 mg/kg clopidogrel daily for 7 days, and then anesthetized with isoflurane. The distal 4 mm of their tails were then transected, and the tails were submerged in saline that was warmed to 37°C. Bleeding from the tails was monitored and the duration to bleeding cessation was recorded until a maximum of 20 minutes. The accumulated hemoglobin content in the saline was then measured using the Drabkin reagent (Sigma-Aldrich) and normalized to a hemoglobin standard (Sigma-Aldrich) by measuring absorbance at 540 nm.

### Platelet isolation

Mice were anesthetized with isoflurane and whole blood was collected by cardiac puncture. The collected blood was immediately mixed with an acid-citrate-dextrose (1.5% citric acid, 2.5% trisodium citrate, and 2% dextrose) solution (9:1) and further diluted 3 to 4 times with modified Tyrode’s buffer [134 mM NaCl, 2.9 mM KCl, 0.34 mM Na_2_HPO_4_, 12 mM NaHCO_3_, 20 mM HEPES, 1.0 mM MgCl_2_, 5.0 mM glucose, and 0.2% BSA (pH 7.35)]. Whole blood was centrifuged at 100*g*, without brake for 15 min at room temperature. Platelet-rich plasma (PRP) was carefully collected, and to inhibit platelet activation, prostaglandin E1 (1 μM) was added immediately. PRP samples were centrifuged at 700*g* for 5 minutes to obtain platelet pellets. Pellets were then washed with modified Tyrode’s buffer in the presence of prostaglandin E1 (1 μM) and lastly resuspended in modified Tyrode’s buffer. For some experiments, platelets were isolated and cultured using a citrate buffer without dextrose and Tyrode’s buffer with 0 mM glucose. After isolation, platelets were treated with saline or 10 mM 2-DG for 30 minutes.

### Flow cytometry

For neutrophil 2-NBDG uptake and total ROS quantification, the procedure described above for human neutrophils was followed. To evaluate platelet P-selectin expression, washed platelets (1×10^8^) were stimulated with and without convulxin (50 ng/mL, Santa Cruz Biotechnology sc-202554) or PAR4 agonist peptide (50 μM, Sigma-Aldrich A3227) for 15 minutes and fixed with 2% paraformaldehyde. Next, platelets were labeled with PE-conjugated anti-CD62P (Biolegend, 148306) and FITC-conjugated anti-CD41 antibodies (Biolegend, 133903) at 1:50 dilution and fluorescence was measured using a BioRad-ZE5 flow cytometer.

### In vivo venous thrombosis

To model large-vein thrombosis, we employed an electrolytic model that has been used previously by our group and others (20, 83). Briefly, after exposure of the IVC, any lateral branches were ligated using 7–0 Prolene suture (back branches remained patent). A 30-gauge silver-coated copper wire (Electrospec, KY-30-1-GRN) with exposed copper wire at the end was placed inside a 25-gauge needle and inserted into the IVC where it was positioned against the anterior wall (anode). Another needle was implanted subcutaneously (cathode), completing the circuit. A constant current of 250 μA was applied for 15 minutes. The current was supplied by a voltage-to-current converter that we described in detail previously (83). After removal of the needle, the abdomen was closed. Before recovery from anesthesia, mice received a single intravenous injection of either control or APS IgG (500 μg). 24 hours later, mice were humanely euthanized, blood was collected, and the resulting thrombus was excised and measured.

### Quantification of MPO-DNA complexes

MPO-DNA complexes were quantified similarly to what has been previously described (84). This protocol used several reagents from the Cell Death Detection ELISA kit (Roche, 11544675001). First, a high-binding EIA/RIA 96-well plate (Costar) was coated overnight at 4°C with anti-human MPO antibody (Bio-Rad, 0400-0002), diluted to a concentration of 0.5 μg/mL in coating buffer (Cell Death kit). The plate was washed 3 times with wash buffer (0.05% Tween 20 in PBS), and then blocked with 1% BSA in PBS for 1 hour at room temperature. The plate was again washed 3 times before incubating for 1 hour at room temperature with 1:500 mouse serum in the aforementioned blocking buffer. The plate was washed 5 times, and then incubated for 1 hour at room temperature with 10× anti-DNA antibody (HRP-conjugated; Cell Death kit) diluted 1:100 in blocking buffer. After 5 more washes, the plate was developed with 3,3′,5,5′-TMB substrate (Invitrogen) followed by a 2N sulfuric acid stop solution. Absorbance was measured at a wavelength of 450 nm with a Synergy HT Multi-Mode Microplate Reader (BioTek).

### Thrombus sectioning and IHC

Isolated thrombi were formalin fixed and paraffin embedded according to standard protocols and stained with H&E. For IHC, 4-μm sections were deparaffinized and rehydrated with standard xylene-to-ethanol washes. Heat-induced epitope retrieval was achieved by boiling samples for 30 minutes in sodium citrate buffer (10mM sodium citrate, 0.05% Tween 20, pH 6.0). Samples were then blocked for 1 hour in PBS with 10% FBS and 0.025% tween. Sections were incubated overnight at 4°C with purified Rat Anti-Mouse Ly6G (BD Biosciences, 551459). After rinsing in PBS, endogenous peroxidases were quenched using 0.3% H_2_O_2_ in PBS for 15 minutes. Samples were then incubated with HRP-conjugated donkey anti-rabbit secondary antibody (Jackson ImmunoResearch, 711-035-152) for 1 hour at room temperature. Color change was detected with a DAB Substrate Kit (Abcam, ab64238) for 5 minutes. Images of thrombi were captured with a Cytation 5 Cell Imaging Multi-Mode Reader. ImageJ Software (NIH) was used to quantify thrombus-infiltrating neutrophils by counting the number of Ly6G-positive cells in a representative area of each thrombus section.

### Complete blood counts

Peripheral leukocyte and platelet counts were determined with an automated Hemavet 950 counter (Drew Scientific).

### Statistics

Data analysis was performed with GraphPad Prism software version 8. Data represent mean ± SEM. For continuous variables, group means were compared by either 2-tailed *t* test (2 groups) or 1- or 2-way ANOVA (more than 2 groups) with corrections for multiple comparisons. A *P* value of < 0.05 was considered statistically significant.

### Study approval

The human aspects of the study complied with all relevant ethical regulations and were approved by the University of Michigan IRB (HUM00122519); all participants provided informed consent for blood donation. All mouse experiments were approved by the University of Michigan IACUC (PRO00010049).

### Data availability

Values for all data points in the figures can be found in the Supplemental Supporting Data Values file.

## Author contributions

AT, YZ, AHS, CAL, and JSK designed the study. AT, AH, SKN, MDR, CER, KS, JH, GS, SY, SKE, CKH, TS, and CS conducted experiments and acquired data. JAM, JKS, and SLS identified patients for recruitment. AT, AH, SKN, and JSK analyzed data. AT and JSK wrote the manuscript, which was critically revised and then approved by all authors before submission.

## Supplementary Material

Supplemental data

Supporting data values

## Figures and Tables

**Table 1:**
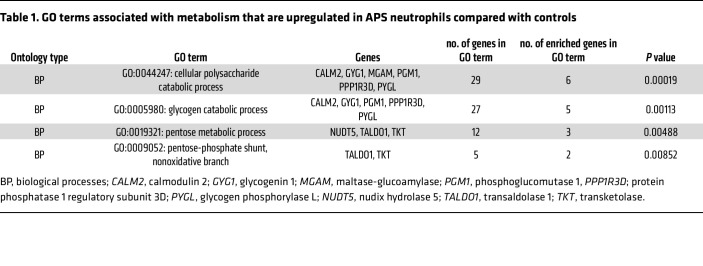
GO terms associated with metabolism that are upregulated in APS neutrophils compared with controls

## Version 1 06/13/2024

In-Press Preview

## Version 2 08/01/2024

Electronic publication
