# Supplementary material for: Neutrophil glucose flux as a therapeutic target in antiphospholipid syndrome
Source: J Clin Invest. 2024 Jun 13;134(15):e169893. doi: 10.1172/JCI169893 (PMC11290966; doi:10.1172/JCI169893)
Supplement: Supplemental data [file jci-134-169893-s048.pdf]

SUPPORTING INFORMATION

Neutrophil glucose flux as a therapeutic target in antiphospholipid syndrome

Ajay Tambralli[1,2], Alyssa Harbaugh[1], Somanathapura K. NaveenKumar[1], Megan D. Radyk[3], Christine E. Rysenga[1], Kaitlyn Sabb[1], Julia Hurley[1], Gautam Sule[1], Srilakshmi Yalavarthi[1], Shanea K. Estes[1], Claire K. Hoy[1], Tristin Smith[1], Cyrus Sarosh[1], Jacqueline A. Madison[1,2], Jordan K. Schaefer[4], Suman L. Sood[4], Yu Zuo[1], Amr H. Sawalha[5], Costas A. Lyssiotis[3], Jason S. Knight[1]

Affiliations
[1] Division of Rheumatology, Department of Internal Medicine, University of Michigan, Ann Arbor, Michigan, USA
[2] Division of Pediatric Rheumatology, Department of Pediatrics, University of Michigan, Ann Arbor, Michigan, USA
[3] Department of Molecular and Integrative Physiology, University of Michigan, Ann Arbor, Michigan, USA
[4] Division of Hematology & Oncology, Department of Internal Medicine, University of Michigan, Ann Arbor, Michigan, USA
[5] Departments of Pediatrics, Medicine, and Immunology, and Lupus Center of Excellence, University of Pittsburgh School of Medicine, Pittsburgh, PA, USA

Correspondence
Jason S. Knight, MD, PhD
5560 Medical Science Research Building 2
1150 W Medical Center Drive, Ann Arbor, MI 48109
Telephone: 734-763-3031
jsknight@umich.edu

**Supplemental Table 1**: Participant characteristics				
	**Control**	**APS**	**aPL only**	**Thromb (aPL-)**
**Demographics**				
Number	42	34	9	9
Female	24 (57%)	21 (62%)	8 (89%)	4 (44%)
Male	18 (43%)	13 (38%)	1 (11%)	5 (56%)
White or Caucasian	21 (50%)	33 (97%)	8 (89%)	8 (89%)
Asian	21 (50%)	0 (0%)	0 (0%)	0 (0%)
Race not reported	0 (0%)	1 (3%)	1 (11%)	1 (11%)
Age (years) ± SD	41 ± 15	51 ± 17	49 ± 10	51 ± 20
**Labs**				
Lupus anticoagulant	-	16 (47%)	6 (67%)	0 (0%)
Anti-β_2GPI IgG	0 (0%)	25 (74%)	2 (22%)	0 (0%)
Anti-β_2GPI IgM	0 (0%)	14 (41%)	6 (67%)	0 (0%)
Anticardiolipin IgG	-	24 (71%)	2 (22%)	0 (0%)
Anticardiolipin IgM	-	14 (41%)	5 (56%)	0 (0%)
**Clinical manifestations**				
Any thrombosis	0 (0%)	32 (94%)	0 (0%)	9 (100%)
Arterial thrombosis	0 (0%)	17 (50%)	0 (0%)	1 (11%)
Venous thrombosis	0 (0%)	20 (59%)	0 (0%)	8 (89%)
Pregnancy morbidity	0 (0%)	3 (9%)	0 (0%)	0 (0%)
Small vessel thrombosis	0 (0%)	6 (18%)	0 (0%)	0 (0%)
CAPS	0 (0%)	4 (12%)	0 (0%)	0 (0%)
DAH	0 (0%)	2 (6%)	0 (0%)	0 (0%)
APS nephropathy	0 (0%)	3 (9%)	0 (0%)	0 (0%)
**Medications**				
Warfarin	0 (0%)	18 (53%)	0 (0%)	0 (0%)
Low-molecular-weight heparin	0 (0%)	9 (26%)	0 (0%)	0 (0%)
Direct thrombin inhibitor	0 (0%)	0 (0%)	0 (0%)	0 (0%)
Factor Xa inhibitor	0 (0%)	3 (9%)	0 (0%)	9 (100%)
Aspirin	0 (0%)	12 (35%)	7 (78%)	1 (11%)
Hydroxychloroquine	0 (0%)	22 (65%)	5 (56%)	0 (0%)
Immunosuppressant	0 (0%)	8 (24%)	2 (22%)	0 (0%)

aPL: antiphospholipid antibody, APS: antiphospholipid syndrome, CAPS: catastrophic antiphospholipid syndrome, DAH: diffuse alveolar hemorrhage. Immunosuppressants include azathioprine, leflunomide, methotrexate, mycophenolate, or rituximab.

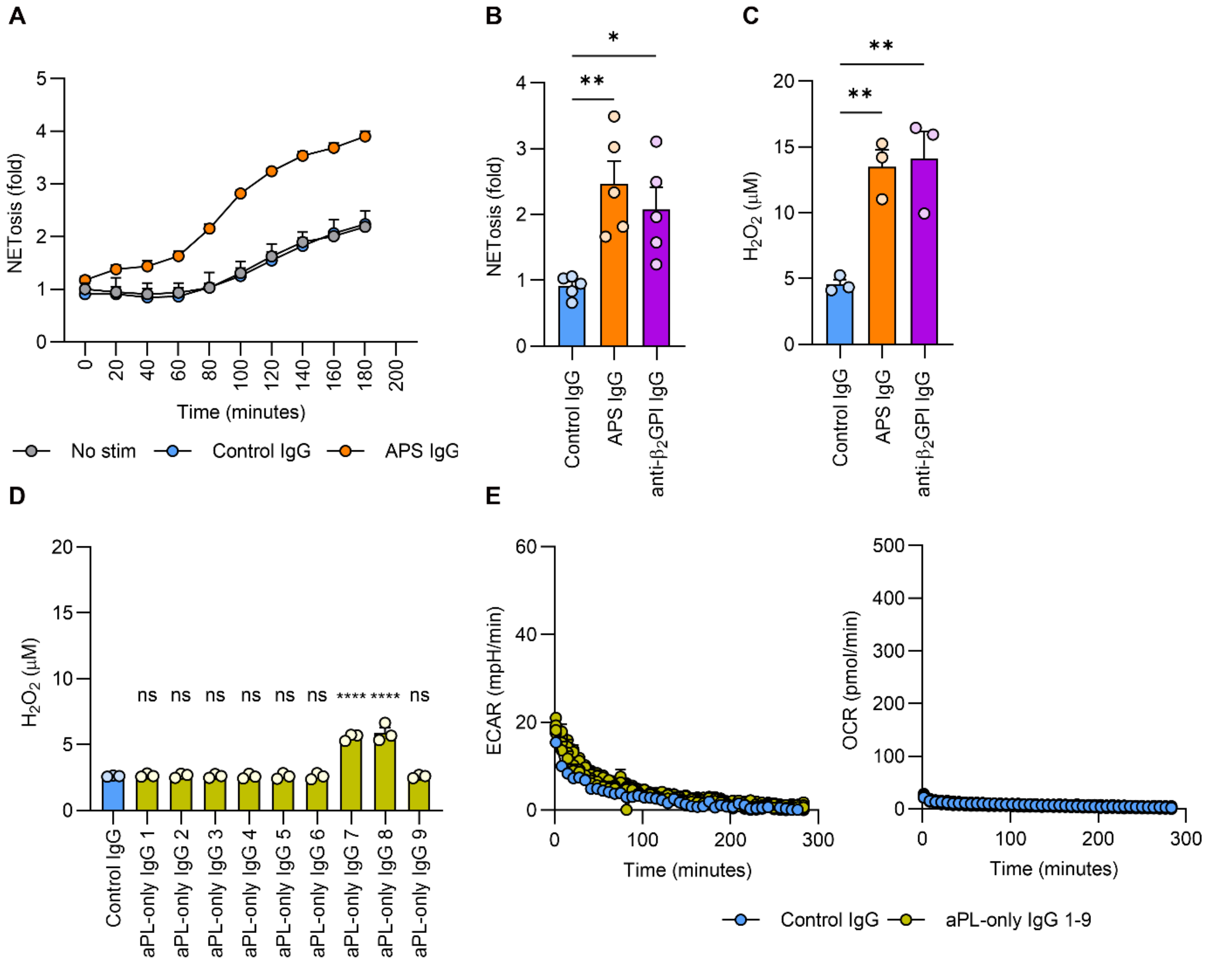

Supplemental Figure 1. (**A**) Neutrophils from controls were treated with PBS (no stim), control IgG (10 μg/mL), or APS IgG (10 μg/mL). NETosis was monitored by measuring SYTOX Green fluorescence every 20 minutes for 3 hours. Fluorescence was normalized to the PBS-treated neutrophils at time 0. Data are representative of 3 independent experiments. (**B**) Neutrophils from controls (n=5) were treated with control IgG (10 μg/mL), APS IgG (10 μg/mL), or affinity-purified anti-β_2GPI IgG (10 μg/mL) for 3 hours, and NETosis was quantified using SYTOX Green. All data are presented as fold change compared with neutrophils that were not treated with any stimuli; *$p<0.05$ and **$p<0.01$ using one-way ANOVA with Holm-Sidak multiple comparison test. (**C**) Neutrophils from controls (n=3) were stimulated as in (B) for 1 hour, and cytosolic ROS production was quantified using the Amplex Red reagent; **$p<0.01$ using one-way ANOVA with Holm-Sidak multiple comparison test. (**D**) Neutrophils from controls (n=3) were stimulated with control IgG (10 μg/mL) or individual IgG fractions (10 μg/mL) from the nine aPL-only patients for 1 hour, and cytosolic ROS production was quantified using the Amplex Red reagent; comparisons are to control IgG-treated neutrophils and ****$p<0.0001$ using one-way ANOVA with Holm-Sidak multiple comparison test. (**E**) Using a metabolic flux analyzer, neutrophils from controls were treated with control IgG or each of the nine aPL-only IgG fractions. ECAR (left) and OCR (right) trends were measured over 4 hours. These data are representative of 3 independent experiments.

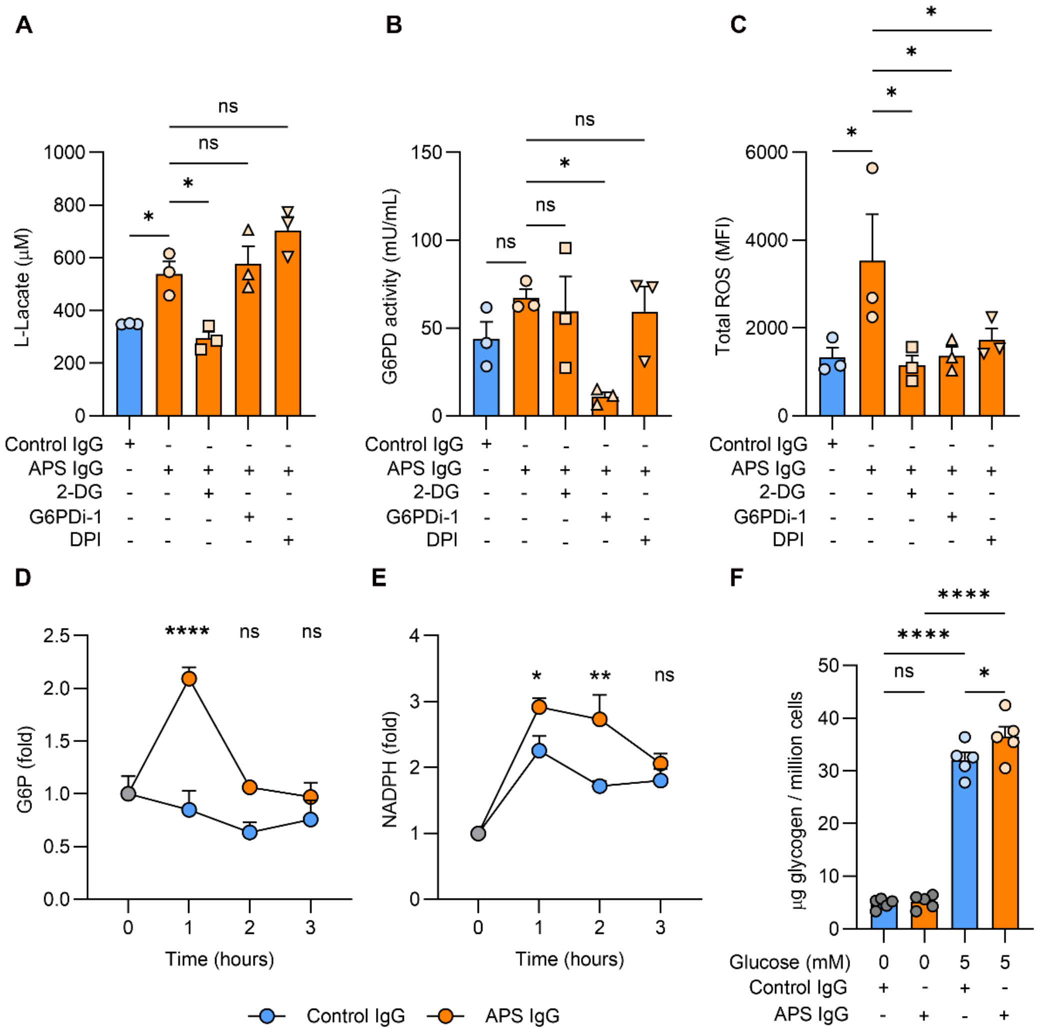

Supplemental Figure 2. Metabolic parameters for APS IgG-induced neutrophil activation. For **A-C**, neutrophils from controls (n=3) were treated with PBS, 2-DG (10 mM), G6PDi-1 (50 μM), or DPI (10 μM) for 30 minutes and then stimulated with control IgG (10 μg/mL) or APS IgG (10 μg/mL) for 2 hours. Then, (**A**) L-Lactate in cell culture supernatant was measured with a colorimetric assay, (**B**) intracellular G6PD enzyme activity was measured with fluorometric assay, and (**C**) total cellular ROS production was measured with DCFDA fluorescence using flow cytometry. For A-C, all statistical comparisons are to APS IgG neutrophils treated with PBS (i.e., no inhibitor); *p<0.05 and ns=not significant using one-way ANOVA with Holm-Sidak multiple comparison test. For **D-E**, neutrophils from controls (n=3) were incubated for 3 hours with control or APS IgG and every hour (**D**) intracellular G6P and (**E**) NADPH were quantified. Data are normalized to the values obtained at 0 hour without any IgG treatment. Comparisons are shown for each time point; *p<0.05, **p<0.01, ****p<0.0001, and ns=not significant using two-way ANOVA with Holm-Sidak multiple comparison test. (**F**) Neutrophils from controls (n=5) were incubated in media with and without glucose as indicated and intracellular glycogen was measured after 2 hours. *p<0.05, ****p<0.0001, and ns=not significant using one-way ANOVA with Holm-Sidak multiple comparison test.

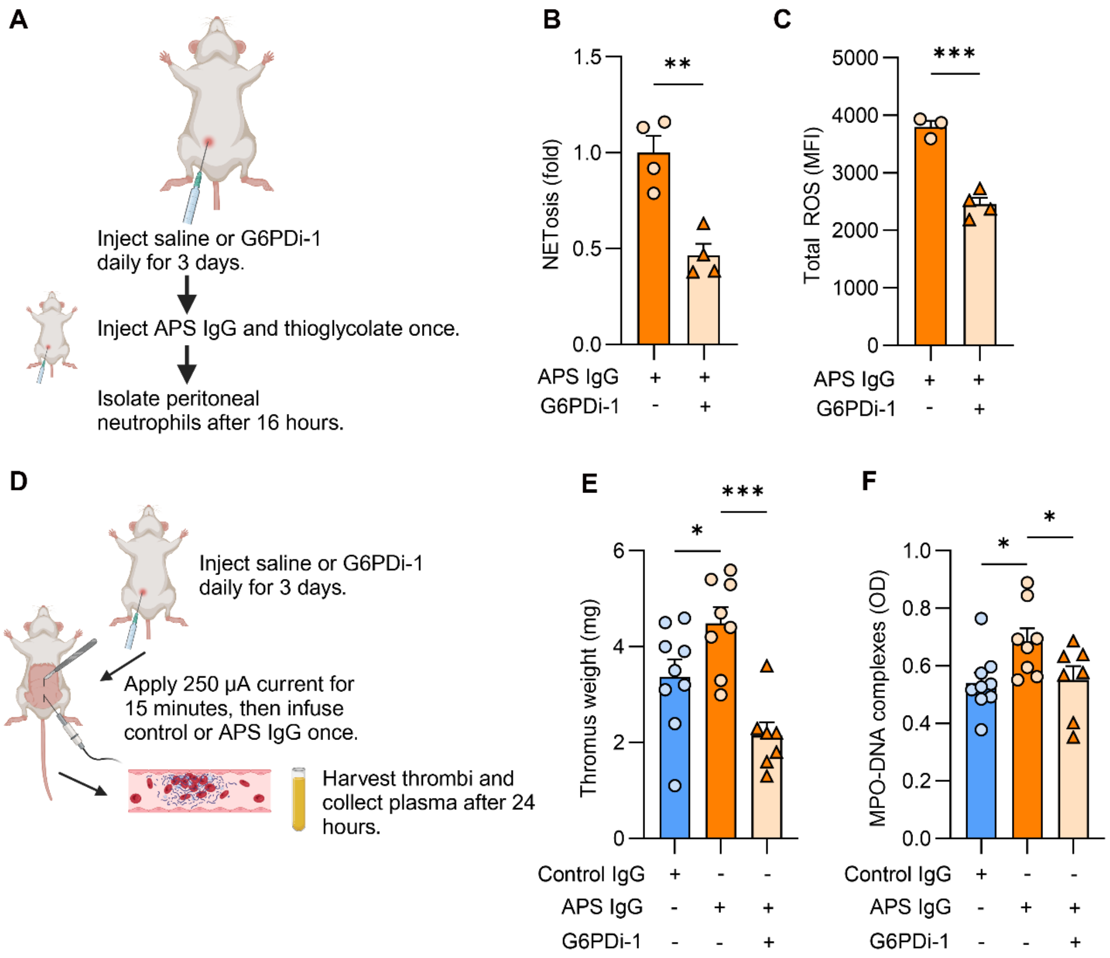

Supplemental Figure 3. G6PDi-1 restrains NETosis and mitigates APS IgG-induced thrombosis in mice. (**A**) Timeline of treatment with saline (n=3-4) or G6PDi-1 (25 mg/kg, n=4), intraperitoneal IgG administration, and peritoneal neutrophil isolation. Each point represents one mouse. (**B**) Spontaneous NETosis was characterized using SYTOX Green. Data are presented as fold change in the neutrophils from APS IgG + G6PDi-1-treated mice as compared with APS IgG + saline-treated mice; **p<0.01 using t-test. (**C**) Total cellular ROS production was measured with DCFDA fluorescence using flow cytometry; ***p<0.001 using t-test. (**D**) Timeline of treatment with saline or G6PDi-1 (25 mg/kg) and schematic of APS IgG-induced electrolytic injury model; n=9 for control IgG + saline, n=8 for APS IgG + saline, and n=7 for APS IgG + G6PDi-1. (**E**) Thrombus weights and (**F**) plasma MPO-DNA complexes were measured; *p<0.05 and ***p<0.001 using one-way ANOVA with Holm-Sidak multiple comparison test.
